# Supplementation of Diets With Bovine Colostrum Influences Immune and Gut Function in Kittens

**DOI:** 10.3389/fvets.2021.675712

**Published:** 2021-08-10

**Authors:** Asa M. Gore, Ebenezer Satyaraj, Jeff Labuda, Robyn Engler, Peichuan Sun, Wendell Kerr, Lisa Conboy-Schmidt

**Affiliations:** Nestlé Purina Research, Saint Louis, MO, United States

**Keywords:** GALT, gut-associated lymphoid tissue, bovine colostrum, nutritional immunology, gut microflora, gut health

## Abstract

In its early life a kitten faces many significant events including separation from its mother, re-homing and vaccination. The kitten is also slowly adapting to their post-weaning diet. Recent advances in companion animal nutrition have indicated that functional ingredients such as colostrum can help support the immune system and gastrointestinal health. Here we report for the first time the effect of feeding a diet containing 0.1% spray dried bovine colostrum (BC) to growing kittens on gut-associated lymphoid (GALT) tissue responses, systemic immune responses, and on intestinal microbiota stability. BC supplementation induced increased faecal IgA expression, and a faster and stronger antibody response to a rabies vaccine booster, indicative of better localised and systemic immune function, respectively. BC supplementation also helped to maintain kittens' intestinal microbiota stability in the face of a mildly challenging life event. These results show that BC supplementation can help strengthen the immune system and enhance the gut microbiota stability of growing kittens.

## Introduction

Kittens encounter many new and challenging experiences during their first year of life (weaning, vaccination, re-homing etc.). These challenges may adversely affect their health. Stress can suppress the immune system and lead to alterations in the gastrointestinal (GI) tract. Given the number of new experiences faced by a young kitten and the immature nature of the kitten's immune system, it is not surprising that the post-weaning period is associated with increased risk of infection (1) and GI upset (2). For example, an analysis summarizing post-weaning feeding problems in young kittens that were 1–4 mos. of age showed that 55% suffered from infectious diseases with the most common being parvovirus, herpesvirus, and calicivirus (1). The GI tract has protective functions that help to prevent the invasion of pathogens and neutralize toxins. There have been limited *in vivo* studies regarding the kitten neonate GI tract and immune functionality. For possible comparison, the mucosal secretions of the neonatal piglet GI tract contains very low levels of IgA due to low levels of Ig containing cells, with IgA production increasing with the development of IgA positive B cells at about 5 weeks (3). IgA, the most abundant immunoglobulin produced by the GALT (Gut Associated Lymphoid Tissue), plays a key role in excluding antigens from entering the epithelium and in the selection and maintenance of colonizing bacteria (4). IgA also neutralizes toxins and viruses. The maturation of the gut immune system after birth is highly influenced by the presence of bioactive components present in the mother's colostrum and milk (5), and modulated by the introduction of solid food (3).

The GI microbiota also serves a critical role in the development of the host animal's immune system (6, 7). Within a day or two of birth, the new-born's entire digestive tract is populated with microorganisms from the environment, with transfer from the mother's colostrum as the dominant source (8). Although studies in kittens and cats in general are limited, a recent study by Jia *et al*. found that kitten microbiota profiles were more diverse at 4 weeks (unweaned) than at 4 and 9 months (weaned), suggesting maturation to a more stable profile somewhere between 4 weeks to 4 months (9). Interestingly, Jia *et al*. demonstrated that the composition of the kitten's GI microbiota was modulated by diet (9). The ability of pathogenic or beneficial organisms to become established is much greater during periods of microbial transition (6, 10). Clearly, the post-weaning period in the growing kitten is a time when optimal nutrition can benefit the immune system and microbiota development.

Colostrum is the milk produced shortly after birth which meets the unique nutritional needs of new-borns, and which also transfers passive immunity and promotes the growth and development of the GI tract (5). Colostrum has attracted significant interest as a nutritional strategy for immunomodulation in adults (11, 12). We very recently demonstrated that bovine colostrum (BC) supplementation improves gut immunity and gut health, as well as targeted immune responses in adult dogs (13). Another study demonstrated improvement in fecal quality associated with feeding BC to puppies (14). BC has been proven to contain bioactives including immunoglobulins, lactoferrin, immune cells such as neutrophils, macrophages, and cytokines (15, 16), and also growth factors such as epidermal growth factor (EGF), insulin-like growth factor-1 and 2 (IGF-1 and 2), platelet-derived growth factor (PDGF) and transforming growth factor-β (TGF-β) (17). Colostrum has been widely reported to be immunostimulatory and has disease preventing properties (11, 12), with effectiveness against disorders of the GI tract. To our knowledge, there are no published reports on the effect of feeding BC to kittens in the post-weaning period. The aim of this study was to investigate BC's immunomodulatory and gut stabilisation effects in weaned and growing kittens.

## Materials and Methods

### Animals and Diets

Twenty-four domestic shorthair kittens from 8 litters were weaned at 12 weeks of age (week−4; kittens ages 12 weeks) and then fed a pre-test complete and balanced kitten food for 4 weeks [Nestlé Purina Product: approximately 40% protein, 21% fat, 21% carbohydrate, 0.7 Crude fiber; metabolizable energy 16401.28 kJ/kg (3920 kcal/kg)]. At the end of the 4-week pre-test period, (week 0; kittens aged 16 weeks), baseline measurements were taken, and kittens were blocked into diet groups based on litter, weight, age and sex. At this time, all kittens were vaccinated with a rabies vaccine (IMRAB® 3, Merial Inc, Duluth, GA) as part of their normal veterinary care. A booster rabies vaccine was given at week 38. BC was incorporated into the diet by taking the dry powder and mixing it with vitamins, minerals, and palatants to form a post-extrusion premix that was then spray-dried onto the control diet. Half the kittens were fed a ‘Control' diet which was the same as the pre-test diet and the other half fed the ‘Test diet' which was the control diet supplemented with 0.10% spray-dried BC (Sterling Technology Inc., Brookings, SD). Experimental diets were produced at the Nestlé Purina Pet Care pilot manufacturing facility, St. Louis, MO and fed to completion of the study. Kittens in each dietary group were housed separately with ad-libitum access to food and water. Kittens were fed twice per day, once in the morning and once in the evening. Food intake was recorded daily, and body weight was recorded weekly. During the period from weeks 12–20, the kittens remained with their litters and in the rooms they were born in. At 20 weeks of age (week 4 of the study), kittens were relocated from the nursery to open room housing (this event was considered a challenging event). The trial protocol was conducted in strict accordance with the guidelines established by the Nestlé Purina Pet Care Advisory Committee and approved by the Nestlé Purina animal care and use committee.

**Figure 1:**
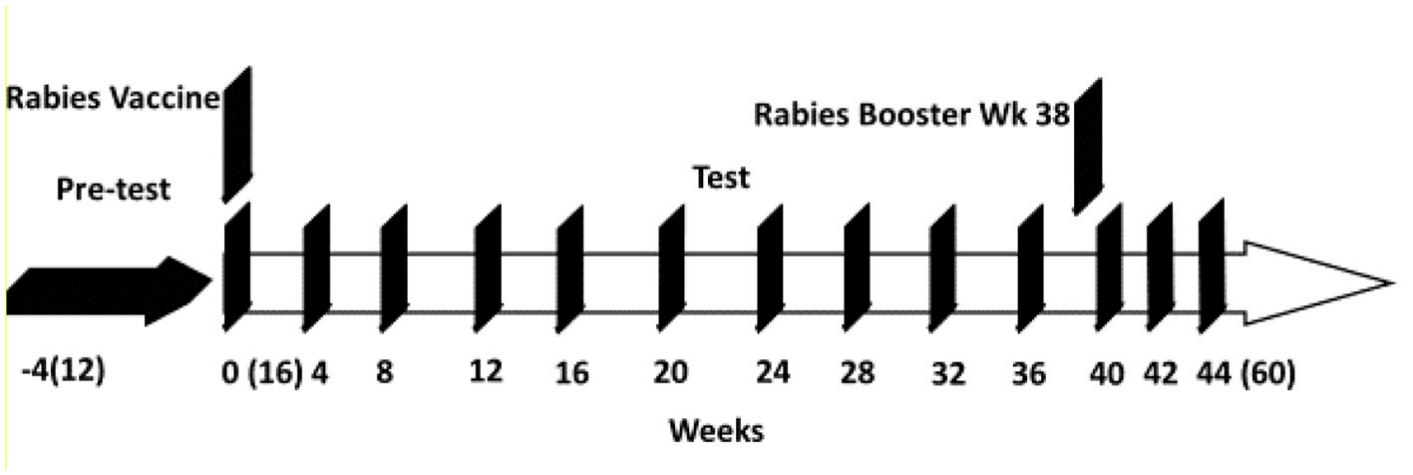
Study schematic for kitten study from weeks −4 to 44. Kitten ages from (12) weeks old to (60) weeks old.

Jugular or femoral blood samples were collected at weeks 0, 4, 8, 12, 16, 20, 24, 28, 32, 36, 40, 42, and 44 (using BD Vacutainers with sodium citrate as the anticoagulant, Becton & Dickenson). To obtain plasma, blood samples were centrifuged at 10,000 *RCF* for 10 min at 6°C and plasma stored at −80°C until assayed for immune markers. Fecal samples were collected at weeks −4, 0, 4, 12, 16, 24, 32 and 40, and immediately stored in a −80°C freezer. Body weights of the kittens were recorded weekly. A schematic diagram for the trial is shown in Figure 1.

### Measurements of Antibodies in Plasma

A Rapid Fluorescent Focus Inhibition Test (RFFIT) was used to measure serum rabies virus neutralizing antibodies. This is a functional assay and measures the ability of antibodies in the serum to neutralize rabies virus, and hence is a good reflection of how effectively the kitten in question would be able to ward off a potential infection with the rabies virus. The RFFIT test was only measured from blood samples collected at weeks 36, 40, 42 and 44. The test was carried out by the Rabies Laboratory of Kansas State University, Manhattan Kansas, USA according to a method previously published (18).

### Measurements of Antibodies in Faeces

Secretary IgA levels in faecal samples were measured as an indicator of GALT activity. Fecal samples from weeks −4 to 40 were used to assess secretory IgA. Using 1.5 ml of the protein extraction buffer (50 mM-EDTA and 100 mg/l soybean trypsin inhibitor in PBS/1% bovine serum albumin from Sigma, St Louis, MO), 0.5 g of faeces were vortexed. Phenylmethanesulphonyl fluoride (50ml, 350 mg/l from Sigma) was added to each tube, and the samples were centrifuged at 10,000 *RCF* for 20 min. at 4°C. The supernatants were collected and frozen at−80°C until assayed for IgA by ELISA as follows: a 96-well plate was coated overnight at 4°C with a 1/100 dilution of mouse anti-feline IgA (Serotec, Raleigh, NC) in 50 μl borate buffer and then washed with PBS-0.1% Tween 20. Free binding sites were blocked with 100 μl of PBS containing 5% foetal calf serum and 0.1% Tween 20 (ELISA buffer) for 1 hour at 37°C. Duplicate faecal protein extracts were placed in the wells and incubated for 2 hour at 37°C and then washed several times with PBS-Tween 20. The plate was incubated with a 1/10,000 dilution of polyclonal goat anti-feline IgA conjugated with HRP (Serotec, AAI31P) in ELISA buffer (final volume 50 μl) for 1 h at 37°C and washed with PBS-Tween 20. Colour development was done with 50 μl of 3, 3,′ 5, 5′-Tetramethylbenzidine (TMB) peroxidase substrate (KPL, 50-76-00) according to the manufacturer's instructions. The reaction was stopped with 50 μl of 1 M phosphoric acid. Colour development was read at 450 nm and results were expressed as μg/ml using a feline IgA standard. Values for faecal IgA were normalized with the total protein content. The total protein content was measured using a BCA Protein Assay Kit (Pierce, 23225).

### Measurement of Serum Amyloid A

Serum Amyloid A (SAA) is an acute phase protein that is produced by the liver in response to inflammation (19)_._ SAA was measured as a general marker of inflammation to confirm that immune enhancement was not a result of or did not lead to a generalized inflammatory condition. Serum SAA level was measured in all cats toward the end of the trial at weeks 28, 32, 36, 40, 42, and 44 using a feline SAA kit [Tridelta, Maynooth, Ireland], as per manufacturer's instructions.

### Measurement of Temporal Temperature Gel Electrophoresis (TTGE)

At week 4, kittens experienced a significant life event. Measuring TTGE allowed an assessment of gut microbiota stability before and after this new life challenge or ‘stress' event. This challenge event had 2 components: (1) both groups of kittens were relocated from their nursery rooms to open rooms and (2) kittens were immediately taken from the open rooms to the onsite veterinary clinic for week 4 blood and faecal sampling. Several studies have documented stress experience in cats associated with vet visits, including Tateo et al. (20) who used various behavioural and clinical criteria to confirm that veterinary examination is stressful for cats. Weaning, restraining, handling, veterinary care, parasites, transporting, and heat or cold are all regarded as stress or stress events that can negatively affect performance of farm animals (21). These unfavourable changes include: immune function, which may increase susceptibility to disease, decreased food intake and growth, and decreased fertility (21). Dog and cats are subjected to the same types of life events; therefore components 1 and 2 represented normally occurring challenges which allowed measurement of the effect of the diets on gut microbiota stability. Gut microbiota stability was assessed by TTGE (13). TTGE analysis allows the separation of 16S rRNA gene fragments that have been amplified by PCR and is a commonly used technique to identify microbiota microbial profiles. TTGE analysis was carried out following challenge components 1 and 2 at week 4, and after component 2 only at weeks 24 and 40. For TTGE analysis at weeks 4, 24 and 40, faecal loop samples were taken at various periods, including on −1 Day (before the challenge), 0 pre-challenge (just before the challenge), 0 post-challenge (4 hrs. after challenge), +1 Day (1 day after the challenge), +2 Day (2 days after the challenge), and +3 Day (3 days after the challenge). Samples were immediately frozen at −80°C for later TTGE analysis.

### TTGE Procedure

Genomic DNA from the rectal loops was obtained using a modified extraction method described by Tsai and Olsen (22). Modifications consisted of removing 1 ml lysate for DNA extraction and precipitation of DNA at −80°C for at least 16 hours. TTGE was performed on the extracted DNA according to the method previously published (13). Briefly, TTGE was performed using a Bio-Rad D-code™ system. PCR fragments were separated on 10% polyacrylamide denaturing gels (7M urea). Bacterial standard ladders were created by individual PCR amplifying DNA extracted from predominant intestinal strains and combining the PCR products. Primers used for the construction of the ladder were labeled “green” (6-FAM). The ladder was loaded together with the sample in each lane and was used to map the gel contours and correct for differences in length in migration within and among gels. Gel images were captured and digitized using the FMBIOII (version 1.1) software (Hitachi). Digitized images were analyzed using the GelCompar II (version 2.0) gel analysis software (Applied Maths). Band classes were established and band densities (based on height and band surface) within each class were tabulated. Each band class contained all the bands that migrated to the same adjusted location on the gels.

The banding pattern of samples collected pre-challenge (−1 Day) were used to characterise baseline species banding pattern. The effect of challenge on gut microbiota was evaluated by assessing the per cent similarity of ‘pre'-challenge (−1 Day compared to 0 pre-challenge) and ‘post'-challenge [−1 Day compared to 0 post-challenge, +1 day, + 2 day and +3 day) TTGE profiles. Similarity scores pre and post challenge of the BC supplemented group were compared with those of the control group. ‘Pre'-challenge measure reflects the normal baseline variability of the gut microflora and post-challenge measure provides an aggregate measure of the stress induced change in the gut microflora pattern over a 3-day period following the stress event.

### Statistical Analysis

A Linear Mixed Model (PROC MIXED) was conducted using SAS [SAS 9.3 (2002–2010) SAS Institute Inc., Cary NC, USA] to test overall differences between groups for all measures. When data were combined for fecal IgA and TTGE, overall means across weeks or periods was used. Dunnett's test was used to adjust for multiple comparisons with control group. For all tests, the level of significant difference was set at *p* < 0.05.

## Results

### General Physiological Status

At the start of the trial the average weight of the kittens in the test group was 2.15 kg and in the control group 2.18 kg. Food intake and body weight did not differ between the two groups during the trial (data not shown). There was no significant difference between control and the BC supplemented diet on all blood chemistry parameters measured (data not shown). Levels of SAA, a marker of inflammation, measured toward the end of trial, were well within the normal range [0.34–3.6 μg/ml (23) in both groups (1.80 ± 0.254 SEM μg/ml for control, and 1.78 ± 0.255 SEM μg/ml in the BC supplemented group)].

### Immune Response in the Gut

**Figure 2:**
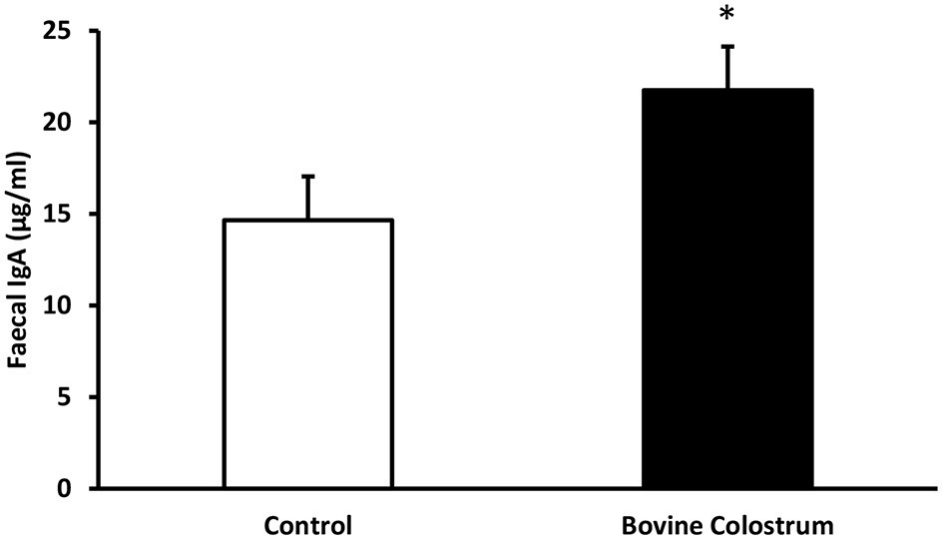
Combined means for IgA levels collected from faecal samples during weeks −4 to 40 from kittens fed diets with or without bovine colostrum. Values are means and vertical bars represent their standard errors (*n* = 12). ^*^Mean value was significantly different from control (*p* < 0.05).

Faecal secretory IgA levels were analysed by ELISA. Fecal IgA values tend to vary quite a bit especially in kittens. Hence, overall changes in the fecal IgA was evaluated over the period of the study rather than weekly changes. Combined means for IgA levels collected from faecal samples during weeks −4 to 40 from kittens fed diets with or without bovine colostrum was calculated. Overall combined average secretory IgA level for the control fed kittens was 14.65 μgm/ml vs. 21.75 μgm/ml for the BC fed kittens. A repeated measures ANOVA demonstrated this was a significant effect of diet on secretary IgA levels, with overall average secretary IgA levels of the BC supplemented diet being 48% higher than those of the control group (*P* < 0.05) (Figure 2). No trends were observed for specific time points, therefore only overall trial means were reported.

### Response to Feline Rabies Virus Vaccine

**Figure 3:**
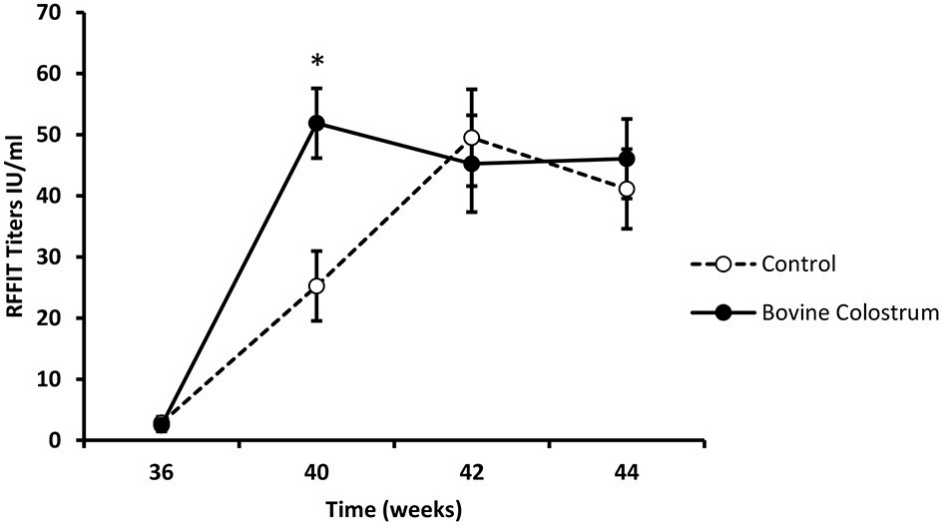
RFFIT antibody titers (IU/ml) in plasma samples collected at weeks 36, 40, 42 and 44 from kittens fed with or without bovine colostrum. Values are means and vertical bars represent their standard errors (*n* = 12). ^*^Mean value was significantly different from control at indicated time point (*p* < 0.05).

All kittens received a rabies vaccination at week 0. Kittens received a booster vaccination at weeks 38 and immune response was evaluated using week 36 as baseline. In our experience with nutritional immunomodulation studies, it takes several weeks before we can detect the impact of the diet on the immune system. Hence, we focused our analysis on the rabies titers following the booster shots, by which time these kittens were on the immune modulating diets for over 30 weeks. It is also for the same reason; we did not measure titers after the first rabies vaccine dose. Repeated measures ANOVA analysis for antibody response at weeks 36, 40, 42 and 44, revealed a significant interaction between the diet and time (*P* < 0.05). In comparison to the control diet, the BC supplemented group had significantly higher antibody levels at week 40 (Figure 3, *P* < 0.01).

### Effects of Bovine Colostrum on Gut Microbiota

**Figure 4:**
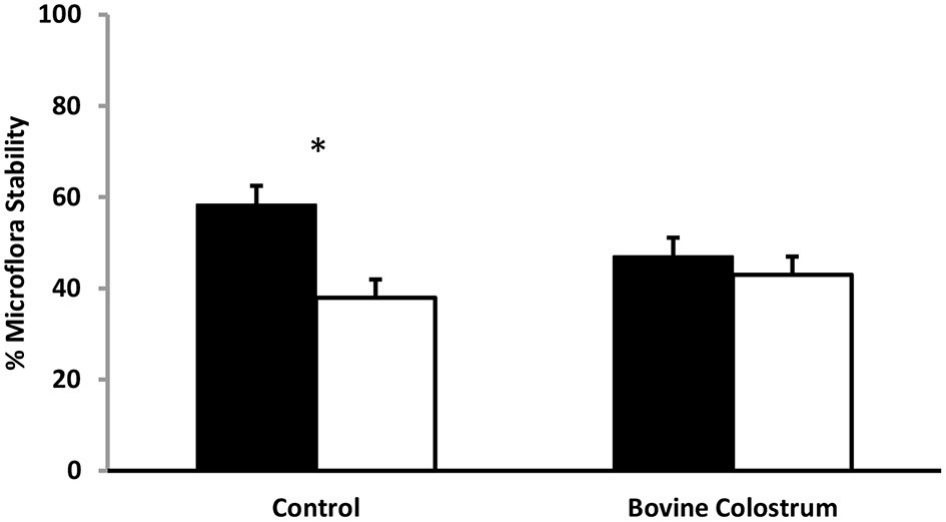
Combined means for TTGE microbial profiling of kittens' gut microbiota pattern for weeks 4, 24 and 40 from kittens fed diets with or without bovine colostrum. Pre-challenge microbiota similarity (banding pattern of −1 day compared to 0 pre-challenge) was compared to post-challenge microbiota similarity (banding pattern of −1 day compared to +1 day) for both groups. Values for each bar are means and vertical bars represent their standard errors (*n* = 12). ^*^Change in Mean value was significantly different for control (*p* < 0.05).

Using TTGE microbial profiling, kittens' gut microbiota patterns were compared before and after a challenging life event. The molecular profile of baseline samples (collected 24 h before challenge) was used for comparison to all other sampling periods. The gut microbiota is dynamic in nature, fluctuating in content over time in response to life events such as stress, age, and illness. Stress can modulate microbiota expression, with a previous study showing that unstressed animals have greater stability of their intestinal bacterial population compared to stressed animals (24). A higher percentage of similarity before and after stress indicates a more stable microbiota population (25). In bovine colostrum and control fed kittens, microbiota stability was compared before and after a challenge and an effect of sampling period was found (*P* < 0.01). Pre-challenge microbiota similarity (banding pattern of −1 day compared to 0 pre-challenge) was compared to post-challenge banding pattern (−1 day compared to +1 day) for BC and control cats. Bovine colostrum supplemented cats had 91% similarity for pre-challenge vs. post-challenge microbiota compared to 65% similarity for the control group (Figure 4, *P* < 0.05). These percentages were combined means for weeks 4, 24 and 40 for each group.

## Discussion

Data collected for body weight gain and general blood chemistry showed that kittens grew normally and had blood chemistry values that were within normal ranges. In addition, SAA levels indicated there was no abnormal inflammatory response due to bovine colostrum addition to the food. These three findings supported that bovine colostrum had no adverse effects on growing kittens.

We have recently shown the immunological benefits of feeding a BC supplemented diet to adult dogs (13). In this current study we extend this finding to kittens showing for the first time, that feeding a complete and balanced kitten food supplemented with bovine colostrum enhances gut immune responses, systemic immune system responsiveness, and microbiota stability. Specifically, BC supplementation was associated with increased faecal IgA production, improved specific immune system response to an innocuous immune challenge i.e. rabies vaccine, and induced greater microbiota stability following a mildly stressful life challenge. These improved indices of gut health and immunological function were not associated with a generalised non-specific hyperactivity of the kittens' immune system, as was measured by SAA.

IgA is the most abundant immunoglobulin produced by the GALT. Therefore, to investigate the effect of bovine colostrum supplementation on the GALT we measured secretory IgA levels in faecal samples. IgA is considered a biomarker of GALT activity (26). Secretory IgA is the form of IgA found in mucosal secretions, including those released to the intestinal lumen. IgA plays a key role in excluding antigens from entering the epithelium and in the selection and maintenance of colonizing bacteria (4). Here we found that faecal secretory IgA levels were significantly enhanced by BC (Figure 2, *P* < 0.05). We recently demonstrated a similar increase in faecal IgA in BC supplemented dogs (13), and in a previous study with dogs supplemented with *Enteroccocus faecium* (SF68) probiotic (27). IgA levels progressively increased over time indicating that continuous feeding of BC has benefits for the growing kitten and may induce a generalised protection against pathogenic infections (28).

The BC supplemented group also demonstrated significantly higher rabies virus neutralizing antibodies, as measured by RFFIT. The BC supplemented group showed a faster and stronger induction of antibody titres, with a statistically significant difference being evident at the week 40 time point (Figure 3, *P* < 0.05). Vaccine responses can be used as clinically relevant biomarkers of an immunological response to challenge, and can be interpreted as a surrogate marker of a typical immune response to infection (12). Studies have shown that human adults who have a poor response to vaccination experience higher rates of clinical illness (29, 30). Interpreting this, the BC supplemented kittens respond stronger and more quickly and would be more likely to mount a more robust immune response against infecting pathogens. BC supplemented kittens did not show evidence of systemic immune hyperactivity. This was evidenced by plasma SAA levels, an acute phase protein that is produced by the liver in response to inflammation (20, 23), being unchanged.

The developed intestinal microbiota contains a relatively stable population of bacteria (25, 31), however, exposure to a challenging life event can induce microbiota instability (24). In this study, we also examined the relative microbiota stability of the BC as compared to control diet and found that a challenging event, in this case either rehousing coupled with blood sampling, or subsequently blood sampling alone, was associated with a reduction in microbiota stability. The similarity before (one day prior to challenge compared to just prior to challenge) and after challenge (one day prior to challenge compared to one day after challenge) for the bovine colostrum fed kittens was 91.3% compared to 65.0% for the control fed kittens (Figure 4, *P* < 0.05). Stressful situations at any age can have a negative impact on immune function (12). Our report here of a mildly stressful life challenge inducing change in kitten microbiota has been similarly shown in mice and rats (24, 32). It is well established that the intestinal microbiota helps protect against pathogenic bacterial colonisation indicating that bovine colostrum supplemented kittens may have an increased protection against infectious pathogens.

Colostrum contains an array of potential immunomodulatory factors (15, 16, 33) that are likely to have contributed to the strengthening of the intestinal and systemic immune systems, and stabilisation of the gut microbiota observed in this study. For example, colostrum contains oligosaccharides which have proven prebiotic actions (34). Oligosaccharides present in human milk act as growth enhancers for *Bifidobacteria* in humans infants (35). BC is also rich in lactoferrin which can inhibit the growth of undesirable microbiota by sequestering iron in the gut (36). In dogs, lactoferrin has been shown to help reduce the faecal content of the pathogenic bacteria *E. coli* and *Clostridium perfringens* (37). In the same study, the authors also showed that lactoferrin had a direct effect on the immune system through increasing blood monocytes, T cells and cytotoxic T cells, and the proliferative response of peripheral blood mononuclear cells. This targeted immune response occurred without an overall change in plasma IgA and IgM (37). Growth factors present in BC such as EGF, TGF-α, TGF-β (38), IGF (39), PDGF, vascular endothelial growth factor (VEGF) and growth hormone (GH) also play an important role in maintaining a healthy gut wall (40).

The colonising intestinal microbiota is critically important in the development of the mucosal immune system (6). For example, the intestinal microbiota is involved in stimulating the proliferation of IgA synthesising plasma cells in the gut (41). Inversely, mucosal IgA also enhances the homeostasis of gut commensal microbiota (42). In this current study, enhanced gut microbiota stability associated with colostrum supplementation is paralleled by increased IgA levels. The stability of gut microbiota and increased IgA faecal content can be reciprocal and indicative of an overall stronger immune system.

Together with our recent finding in adult dogs (13), this current finding of improve localised intestinal immune response, systemic specific immune response, and gut microbiota stabilisation following BC supplementation in kittens, further strengthens the use of bovine colostrum as an immunomodulatory addition to companion animal pet foods.

## Data Availability Statement

The original contributions presented in the study are included in the article/supplementary material, further inquiries can be directed to the corresponding author/s.

## Ethics Statement

The animal study was reviewed and approved by The trial protocol was conducted in strict accordance with the guidelines established by the Nestlé Purina Pet Care Advisory Committee and approved by the Nestlé Purina animal care and use committee.

## Author Contributions

AG and ES designed the study, interpreted the results, and prepared the manuscript. RE, JL, and PS carried out the assays described in the study. AG and ES had primary responsibility for final content. All authors read and approved the final manuscript.

## Conflict of Interest

All authors are employees of Nestlé Purina Research.

## Publisher's Note

All claims expressed in this article are solely those of the authors and do not necessarily represent those of their affiliated organizations, or those of the publisher, the editors and the reviewers. Any product that may be evaluated in this article, or claim that may be made by its manufacturer, is not guaranteed or endorsed by the publisher.

## Abbreviations

BC: bovine colostrum
EGF: epidermal growth factor
GALT: gut-associated lymphoid tissue
GI: Gastrointestinal
IGF-1 and 2: insulin-like growth factor-1 and 2
PDGF: platelet-derived growth factor
TGF-β: transforming growth factor-β
TTGE: temporal temperature gel electrophoresis.
